# Clinical characteristics of narrow-band imaging of oral erythroplakia and its correlation with pathology

**DOI:** 10.1186/s12885-015-1422-7

**Published:** 2015-05-15

**Authors:** Shih-Wei Yang, Yun-Shien Lee, Liang-Che Chang, Cheng-Cheng Hwang, Cheng-Ming Luo, Tai-An Chen

**Affiliations:** 1 Department of Otolaryngology-Head and Neck Surgery, Chang Gung Memorial Hospital, Keelung; No. 222, Mai Chin Road, Keelung, 204 Taiwan; 2 School of Medicine, Chang Gung University College of Medicine, Taoyuan, Taiwan; 3 Genomic Medicine Research Core Laboratory, Chang Gung Memorial Hospital, Tao-Yuan, Taiwan; 4 Department of Biotechnology, Ming Chuan University, Tao-Yuan, Taiwan; 5 Department of Pathology, Chang Gung Memorial Hospital, Keelung, Taiwan

**Keywords:** Erythroplakia, Narrow-band imaging, Carcinoma, Dysplasia, Oral cavity

## Abstract

**Background:**

To analyze the clinical application of endoscope with narrow-band imaging (NBI) system in detecting high-grade dysplasia, carcinoma in situ, and carcinoma in oral erythroplakia.

**Methods:**

The demographic, histopathological data, and NBI vasculature architectures of patients receiving surgical intervention for oral erythroplakia were retrospectively reviewed and analyzed statistically.

**Results:**

A total of 72 patients, including 66 males and 6 females, with mean age of 54.6 ± 11.2 years, were enrolled. The odds ratio of detecting high-grade dysplasia, carcinoma in situ, and carcinoma by twisted elongated morphology and destructive pattern of intraepithelial microvasculature was 15.46 (confidence interval 95 %: 3.81–72.84), and the sensitivity, specificity, positive predictive value, negative predictive value, and accuracy were 80.95 %, 78.43 %, 60.71 %, 90.91 %, and 79.17 %, respectively, which were significantly better than other two established NBI criteria (*p* < 0.001).

**Conclusions:**

Twisted, elongated, and destructive patterns of intraepithelial papillary capillary loop of NBI images are indicators for high-grade dysplasia, carcinoma in situ, and invasive carcinoma in oral erythroplakia.

## Background

Oral cancer incidence has been increasing dramatically over the past few decades, becoming the eighth most common cancer worldwide. The incidence rate of oral cavity cancer is higher for males than for females, and more common in developing than in developed countries [1]. A number of studies have reported that oral squamous cell carcinoma (OSCC) are frequently preceded by or associated with leukoplakia or erythroplakia. Furthermore, it has been shown that leukoplakia and erythroplakia are frequently seen adjacent to some OSCC [2]. The most common potentially malignant disorders include leukoplakia, erythroplakia, lichen planus, and submucous fibrosis. Oral erythroplakia is the rarer form of oral pre-malignant lesion and has been identified as the one with the highest malignant transformation rates [1,3]. However, none of predicting factors of high-grade dysplasia, or carcinoma has been disclosed before the pathology is available.

Flexible fiberoptic endoscope with narrow-band imaging system (NBI) is an advanced optical image enhancement technology that magnified patterns of the surface of mucosa and vessels in the surface of mucosa by employing the characteristics of light spectrum [4]. In addition to the clinical application for detecting precancerous and neoplastic lesions in oropharynx, hypopharynx, larynx, esophagus, stomach, and colon, this newly invented endoscopic technique has been shown to support the evaluation of oral mucosa diseases [5–11]. Histopathologically, erythroplakia commonly shows epithelial change, ranging from dysplasia to invasive carcinoma [12,13]. There is evidence to support the viewpoint that in an individual lesion, the more severe the dysplasia the greater the possibility of it developing into malignancy [14]. Under such circumstances, identification of high-grade dysplasia, carcinoma in situ, and invasive carcinoma (HGD/Tis/CA) within erythroplakia is crucial and beneficial clinically. Our previous works have indicated that the diversiform intraepithelial microvascular morphological patterns detected by NBI are advantageous in identifying HGD/Tis/CA in oral leukoplakia [5,6,8,9]. The aim of this study is to analyze if the current established criteria used to depict neoplastic lesions of the mucosa of the upper aerodigestive tract can also be used in patients with oral erythroplakia, and to evaluate the diagnostic validity of NBI in detecting HGD/Tis/CA in oral erythroplakia.

## Methods

**Fig. 1:**
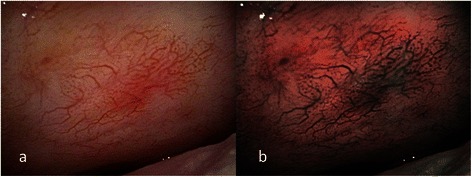
**a** Endoscopic examination of the left buccal oral erythroplakia of a 45-year-old male patient with conventional broadband white light. **b** NBI image from Fig. 1a. Regularly distributed intraepithelial papillary capillary loop was demonstrated on the reddish patch, or IPCL type I, was shown by NBI; the pathological report revealed squamous hyperplasia

**Fig. 2:**
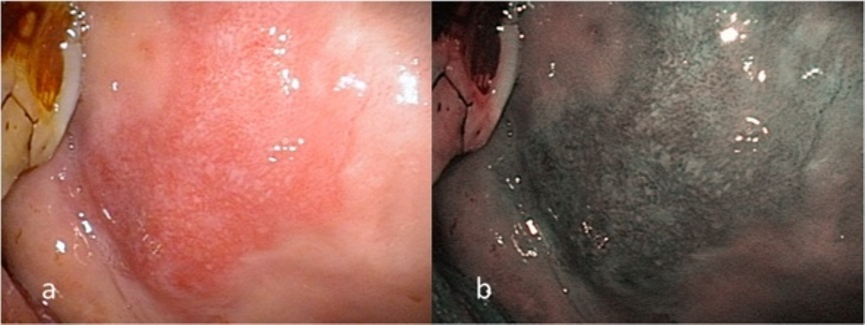
**a** Endoscopic examination of the right buccal erythroplakia of a 48-year-old male patient with conventional broadband white light. **b** NBI image from Fig. 2a. Dilated and tortuous intraepithelial microvasculature, or IPCL type II, was shown by NBI; the pathological report revealed intermediate-grade dysplasia

**Fig. 3:**
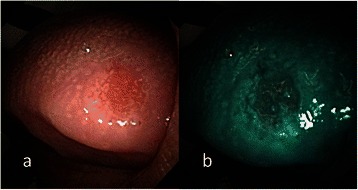
**a** Endoscopic examination of the left tongue erythroplakia of a 70-year-old male patient with conventional broadband white light. **b** NBI image from Fig. 3a. Dilated and tortuous intraepithelial microvasculature, or IPCL type II, was shown by NBI; the pathological report revealed low-grade dysplasia

**Fig. 4:**
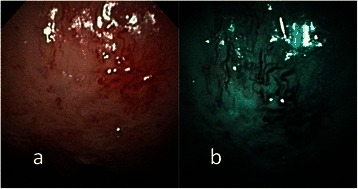
**a** Endoscopic examination of the hard palate erythroplakia of a 64-year-old male patient with conventional broadband white light. **b** NBI image from Fig. 4a. Elongated and twisted intraepithelial microvasculature, or IPCL type III, was shown by NBI; the pathological report invasive squamous cell carcinoma

**Fig. 5:**
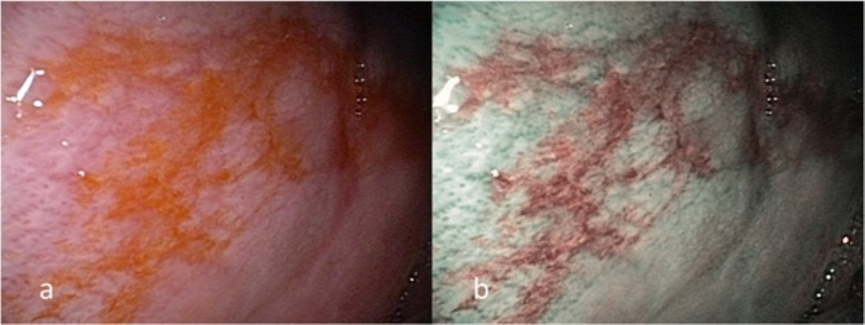
**a** Endoscopic examination of the right buccal erythroplakia of a 65-year-old male patient with conventional broadband white light. **b** NBI image from Fig. 5a. Elongated and twisted intraepithelial microvasculature, or IPCL type III, was demonstrated by NBI, whose pathological report revealed high-grade dysplasia

**Fig. 6:**
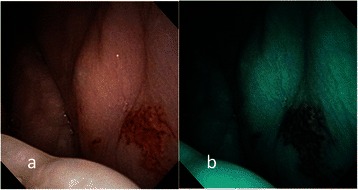
**a** Endoscopic examination of the left buccal erythroplakia of a 39-year-old male patient with conventional broadband white light. **b** NBI image from Fig. 6a. Intraepithelial papillary capillary loop pattern destruction, or IPCL type IV, could be clearly visualized with NBI illumination. The pathological report showed squamous cell carcinoma

**Fig. 7:**
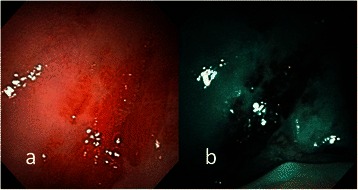
**a** Endoscopic examination of the right retromolar erythroplakia with conventional broadband white light in a 44-year-old male patient with conventional broadband white light. **b** NBI image from Fig. 7a. Destructive pattern of intraepithelial microvasculature, or IPCL type IV, was shown by NBI illumination. The pathological report was squamous cell carcinoma

**Fig. 8:**
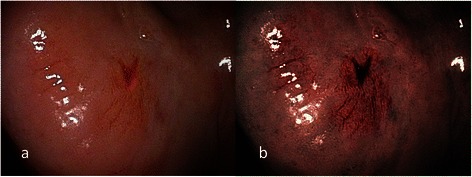
**a** Endoscopic examination of the left ventral tongue erythroplakia with conventional broadband white light in a 56-year-old male patient with conventional broadband white light. **b** NBI image from Fig. 8a. Destructive pattern of intraepithelial microvasculature, or IPCL type IV, was shown by NBI illumination. The pathological report was squamous cell carcinoma

This study was approved by the Institutional Review Board of Chang Gung Memorial Hospital. Records of patients with oral erythroplakia that underwent flexible endoscopy with broad-band white light (BWL) and NBI at the department of otolaryngology of Chang Gung Memorial Hospital, Keelung, from April 2009 to Apr 2012 were retrospectively reviewed. Examinations were carried out with an ENF type V2 and type VQ (Olympus Medical Systems Corp., Tokyo, Japan) NBI endoscope. One light source was utilized as the standard optical filter (BWL) and the other was for the NBI system. The examinations were performed first with BWL illumination with a wide view to observe the whole lesion and its surrounding mucosa. The same procedure was performed with NBI illumination, and the capillaries were analyzed in detail and recorded. The images were recorded and transferred to a hard drive in the computer. Clinical characteristics revealed under BWL were analyzed first, and the intraepithelial papillary capillary loop (IPCL) features under NBI illumination were observed according to the IPCL classification of oral mucosa. The IPCL classification for oral squamous epithelium was created by dividing the findings into type I (normal mucosa, regular brown dots), type II (IPCL pattern dilation and crossing), type III (IPCL pattern elongation and meandering) and type IV (IPCL pattern destruction and angiogenesis following a sequence of carcinogenesis progression) [10]. Written informed consent was signed by every patient, and then total excision by carbon dioxide laser was performed under local anesthesia in the operating room after endoscopic examination. Paraffin-embedded specimens from these patients were collected from the archives of the department of pathology. Hematoxylin- and eosin-stained slides were reviewed by two independent pathologists (L.-C.C. and C.-C.H.) to confirm lesion diagnosis and to determine the final diagnosis. Epithelial dysplasia was diagnosed according to the WHO 2005 classification [14]. Images from NBI were reviewed by two independent otolaryngology specialists (S.-W.Y. and T.-A.C.) to achieve agreement on the morphology of intraepithelial microvasculature. In this study, oral cavity erythroplakia was defined as flat, velvety, sharply demarcated, homogeneous red plaque. Only patients with oral homogeneous erythroplakia were enrolled. Exclusion criteria included oral erythroleukoplakia (mix of both erythroplakia and leukoplakia), non-homogeneous leukoplakia (including speckled, nodular, and verrucous types), reddish exophytic mass with ulceration, and mucosa related to inflammatory or traumatic etiologies. Analysis of the morphology of the microvasculature patterns of oral erythroplakia was performed using the three different reported criteria: (1) criteria I: brownish spots and demarcation line with irregular microvascular patterns (Figs. 1, 2) criteria II: well-demarcated brownish area with thick dark spots and/or winding vessels (Figs. 2, 3), and (3) criteria III: the intraepithelial papillary capillary loop (IPCL) type III (IPCL pattern elongation and meandering, (Figs. 4, 5), and type IV (IPCL pattern destruction and angiogenesis following a sequence of carcinogenesis progression, (Figs. 6, 7, 8) [6,10,15–18]. If more than one IPCL type was detected with NBI, the most advanced type detected was determined as the IPCL type of the lesion. Each patient’s chart records were reviewed, including their demographic data, site of the lesion, morphology of the vascular architecture or the IPCL, and histopathology. Using the histopathological findings as the final diagnostic standards, the odds ratio, sensitivity, specificity, positive predictive value, negative predictive value, accuracy, false positive percentage, and false negative percentage of endoscopy by NBI illumination for detecting HGD/Tis/CA were calculated. Patient histories related to betel quid, alcohol and tobacco use were obtained during our detailed questioning of the patients on their first visit to the otolaryngology clinic of the hospital. The criteria for a positive assignment were defined as previously described [19].

### Statistical analysis

Results are presented descriptively, including factors related to the pathological diagnosis of HGD/Tis/CA which were grouped and analyzed using chi-square test. Odds ratio (OR) and 95 % confidence intervals (CIs) were calculated using a 2-tailed test of significance (*p* < 0.05) for each risk factor. We followed these parameters: (1) when the 95 % CI did not include 1.0, the resulting OR of the risk factor was statistically significant; (2) if the value of the OR was greater than 1.0, the risk was increased, and (3) if the value was less than 1.0, the risk was reduced or protective. The Pearson’s linear correlation coefficient (Pearson’s *r*) between the pathology and endoscopic examination of oral leukoplakia was calculated under the null hypothesis of both samples of pairs showing the same correlation strength [20]. The Fisher’s exact test, ANOVA, and Pearson’s *r* were calculated using the MATLAB program (Mathworks Inc., Natick, Mass., USA).

The predictions and diagnostic tests employed in this study were in accordance with the method described by Simel et al. [21]. The comparison between the two criteria was made on the basis of the changes in the log-odds ratio for the two tables with the standard Pearson chi-square on a 2 × 4 table wherein each row was obtained by treating each 2 × 2 table as a one-way table with four cells. The statistical analyses were also conducted using the MATLAB program.

## Results

The medical records of 72 patients with oral erythroplakia who had received surgical treatment from April 2009 to April 2012 were retrospectively reviewed. Among them were 66 males (91.7 %) and 6 females (8.3 %), whose age ranged from 29 to 83 years, with an average of 54.6 ± 11.2 years. High-grade dysplasia, carcinoma in situ, and squamous cell carcinoma were detected in 21 cases (29.1 %). All four cases of OSCC were stage I, T1N0M0. The demographic and clinicopathological data are shown in Table 1.

**Table 1:** Clinicopathological characteristics of patients with oral erythroplakia (*n* = 72)

Characteristics	Case no. (percent)
Gender	
Female	6 (8.3 %)
Male	66 (91.7)
Age (years)	
54.6 ± 11.2	
Topographic location	
Lip	2 (2.8 %)
Buccal	55 (76.4 %)
Gum	1 (1.4 %)
Tongue	6 (8.3 %)
Palate	2 (2.8 %)
Floor of the mouth	2 (2.8 %)
Retromolar	4 (5.5 %)
Alcohol drinking	
No	29 (40.3 %)
Ex-drinker	23 (31.9 %)
Yes	20 (27.8 %)
Cigarette smoking	
No	14 (19.4 %)
Ex-smoker	19 (26.4 %)
Yes	39 (54.2 %)
Betel quid chewing	
No	21 (29.2 %)
Ex-chewer	36 (50.0 %)
Yes	15 (20.8 %)
History of oral cancer	
No	45 (62.5 %)
Yes	27 (37.5 %)
Intraepithelial microvasculature pattern by NBI	
IPCL type I (regularly distributed arborescent pattern of IPCL)	6 (8.3 %)
IPCL type II (tortuous and dilated pattern of IPCL)	38 (52.8 %)
IPCL type III (twisted and elongated pattern of IPCL)	25 (34.7 %)
IPCL type IV (angiogenesis and destructive pattern of IPCL)	3 (4.2 %)
Pathological diagnosis	
Squamous hyperplasia	12 (16.7 %)
Low-grade dysplasia	22 (30.6 %)
Intermediate-grade of dysplasia	17 (23.6 %)
High-grade of dysplasia/carcinoma in situ	17 (23.6 %)
Squamous cell carcinoma	4 (5.5 %)

*Abbreviation*: *NBI* narrow-band imaging, *IPCL* intraepithelial papillary capillary loop

According to the clinical appearance under endoscopic examination with conventional broadband white light, all of the 72 patients had characteristic oral erythroplakia, including flat, bright red, velvety, often glistening, rather sharply circumscribed, asymptomatic plaque [13,22]. Using NBI illumination, six cases (8.3 %) presented as IPCL type I (Fig. 1), which were all squamous hyperplasia pathologically; 38 cases (52.8 %) as IPCL type II (Figs. 2, 3), squamous hyperplasia in five, low-grade dysplasia in 18, intermediate-grade dysplasia in 11, and high-grade dysplasia in 4; 25 cases (34.7 %) as IPCL type III (Figs. 4, 5), squamous hyperplasia was found in one case, low-grade dysplasia in four, intermediate-grade dysplasia in six, high-grade dysplasia in 13, and squamous cell carcinoma in one; three cases (4.2 %) as IPCL type IV (Figs. 6, 7, 8), all were squamous cell carcinoma. The distribution between the different types of IPCL by NBI system and different pathological results with incremental severity are summarized in Table 2 and Pearson’s linear correlation coefficient was 0.70.

**Table 2:** The case distribution of histopathology among different intraepithelial microvasculature patterns of NBI

	Pathology					Pearson’s linear correlation coefficient
	Squamous hyperplasia	Low-grade dysplasia	Intermediate-grade dysplasia	High-grade dysplasia/Carcinoma in situ	Squamous cell carcinoma	
Morphology of NBI						0.70
IPCL type I	6	0	0	0	0	
IPCL type II	5	18	11	4	0	
IPCL type III	1	4	6	13	1	
IPCL type IV	0	0	0	0	3	

Among the 72 cases in total, 38 met criteria I, with four having HGD/Tis/CA; 63 met criteria II, with 18 having HGD/Tis/CA; and 28 met criteria III, with 17 having HGD/Tis/CA. The odds ratio for criteria I, II, and III were 0.12, 0.80, and 15.46, respectively. The detection rate of HGD/Tis/CA was significantly higher with NBI criteria III than with the other two criteria (*p* < 0.001, Table 3).

**Table 3:** Statistical analysis of pathology, and NBI in detecting high-grade dysplasia, carcinoma in situ, and invasive carcinoma of oral erythroplakia (*n* = 72)

	Non-HGD/Tis/CA^a^	HGD/Tis/CA	Odds ratio (CI 95 %)
Criteria I: Appearance of brownish spots and demarcation line with irregular microvascular patterns			*p* < 0.001*
No	17	17	1.00
Yes	34	4	0.12 (0.03–0.40)
Criteria II: Appearance of well-demarcated brownish area with thick dark spots and/or winding vessels			*p* < 0.001**
No	6	3	1.00
Yes	45	18	0.80 (0.18–3.55)
Criteria III: Elongation, twist, and meandering destruction of IPCL pattern			-
No	40	4	1.00
Yes	11	17	15.46 (3.81–72.84)

*Abbreviation*: *CI* confidence interval

*Comparison between criteria I and criteria III

**Comparison between criteria II and criteria III

^a^Non-HGD/Tis/CA includes squamous hyperplasia, low-grade dysplasia and intermediate-grade dysplasia histopathologically

The sensitivity, specificity, positive predictive value, negative predictive value, accuracy, false positive percentage, and false negative percentage of NBI criteria III for detecting the occurrence of squamous cell carcinoma in oral erythroplakia were 80.95 %, 78.43 %, 60.71 %, 90.91 %, 79.17 %, 21.57 %, and 19.05 %, respectively.

## Discussion

Erythroplakia is a sharply defined, bright red, velvety lesion described by Queyrat in 1911 as occurring on the glans penis and representing a premalignant process, because of its frequent ultimate development of carcinoma [13]. The exact time point for erythroplakia being introduced to describe a specific type of oral mucosa disease is not well documented. A direct relationship between oral erythroplakia and the development of oral cancer was not suggested until the 1960s and the 1970s [22]. Oral erythroplakia has a range of prevalence between 0.02 % and 0.83 %, which is far less than 0.2–4.9 % for oral leukoplakia [22–24]. The term leukoplakia is used to designate a clinical white patch or plaque on the oral mucosa that cannot be removed by scraping and cannot be classified clinically or microscopically as another disease entity [25]. Queyrat used the term “erythroplasie” to designate a red area analogously to the French term “leukoplasie” [22]. The concept of erythroplakia is similar to that of leukoplakia; however, inflammatory or traumatic etiology should be excluded before further diagnosing erythroplakia [26]. In terms of clinical appearance, erythroplakia is different from leukoplakia due to its absence of whitish patch, which was found to be hyperkeratotic lesion under the microscope. Pathologically hyperkeratosis or parakeratosis was not found in cases of oral erythroplakia in the present study. The process of hyperkeratosis is involved in the pathogenic process of leukoplakia, but the same process may not be implicated in erythroplakia. In addition, the epithelium of oral erythroplakia is often atrophic and shows lack of keratinization [1,27]. Erythroplakia has the highest risk of developing carcinoma, whereas this takes place less frequently in oral leukoplakia [28]. In a study done by Shafer et al., 91 % of erythroplakia biopsies revealed dysplasia, carcinoma in situ, or carcinoma pathologically [13]. These distinct differences between these two disease entities are important because the majority of erythroplakia lesions represent precancerous or malignant conditions of more serious magnitude.

Histopathological examination of erythroplakia is the only method that can be used to determine if there is concomitant dysplasia, carcinoma in situ, or carcinoma within erythroplakia, which is also the same for oral leukoplakia. Before a surgical biopsy is conducted, epithelial status is generally not known. NBI is an endoscopic technique based on distinctive optical filters that narrow the light bandwidth to enhance the visualization of the intraepithelial microvasculature of mucosa surface, which rises perpendicularly from the branching vessel, is barely recognizable under observation of normal epithelium by BWL [10,18,29]. It has been shown to be helpful in enhancing early detection of cancerous lesion in the upper aerodigestive tract, including esophagus, pharynx, and oral cavity; abnormal vascular architectures of NBI of oral mucosa appear as increased number, tortuous, dilated, twisted, elongated, and corkscrew-type small blood vessels of varying caliber [5,6,8–10,18,30]. Endoscope with NBI has been employed to evaluate oral cavity leukoplakia, the most commonly seen oral precancerous lesion, and the diversiform intraepithelial microvascular patterns shown by NBI is found to be a useful tool in detecting high-grade dysplasia, carcinoma in situ, and carcinoma in oral leukoplakia in our previous works [5,6,8,9]. No correlation between the clinical appearance of oral erythroplakia and the histopathology has ever been provided so far. Since NBI is characterized by enhancing visualization of the intraepithelial microvasculature, demonstration of microvascular architectures beneath the mucosa epithelium under NBI illumination may elucidate the relationship between the IPCL and pathology of oral erythroplakia.

In the present study, only three cases were IPCL type IV (destructive pattern of intraepithelial microvasculature) and all of them were invasive carcinoma (Figs. 6, 7, 8). Among the 25 cases of IPCL type III (twisted and elongated pattern of intraepithelial microvasculature), 14 cases (56 %) were HGD/Tis/CA. On the contrary, IPCL type I and type II were composed of 44 cases, but only four (9.1 %) were HGD/Tis/CA. The correlation between the different types of intraepithelial microvasculature of NBI and pathological results with step-by-step increased severity was good (Pearson’s *r* = 0.7, Table 2).

Three established criteria of morphology of IPCL of NBI have been utilized clinically. The characteristics of these criteria was investigated and analyzed on oral leukoplakia in our previous work [6]. Criteria I consisted of brownish spots and demarcation line with irregular microvascular patterns, criteria II consisted of well-demarcated brownish area with thick dark spots and/or winding vessels, and criteria III consisted of elongation, twist, and meandering destruction of IPCL pattern. In this study, we further utilized these criteria to analyze and compare the differences in oral erythroplakia. Compared with leukoplakia, the observation of intraepithelial microvasculature is more advantageous because the microvascular morphology of NBI is readily observed (Figs. 1, 2, 3, 4, 5, 6, 7, 8). In the cases of oral leukoplakia, the intraepithelial papillary capillary loop can’t usually be observed owing to the fact that the hyperkeratosis of oral leukoplakia obstructs the penetration of light and the focus of observation has to be emphasized on the mucosa around the whitish patch [5,6,8,9]. It has been suggested that one of the reasons why oral erythroplakia appears red under BWL included attenuated and atrophic epithelium with a vascular lamina propria lying close to the surface and the connective tissue papillae containing engorged capillaries rising between rete ridges close to the surface [13,22]. Significantly increased vascularity with disease progression in oral cancer has been found in a study by Carlie et al*.* In the study it was observed that an alteration in IPCL patterns could be associated with excessive angiogenesis in both premalignant and cancerous lesions [31,32]. The combination of engorged capillaries with increased vascularity accounts for the morphology of microvascular architectures under illumination of NBI. The brownish spots with or without winding vessels had been found to be used in detecting carcinoma in situ in oropharynx and hypopharynx mucosa [18] and cancer lesions in oral cavity [15], however, the low odds ratio of NBI criteria I (OR = 0.12, CI95%: 0.03–0.40) and criteria II (OR = 0.80, CI95%: 0.18–3.55) indicated that these patterns might not be crucial indicators for HGD/Tis/CA in oral erythroplakia. Brownish areas detected by NBI, or criteria I in this study, may represent benign pathologies such as angiodysplasia, erosive changes of the mucosa, or overlapping normal vascularity, each of which may cause false positive results to be observed [33]. The detection rate of HGD/Tis/CA of criteria III (OR = 15.46, CI95%: 3.81–72.84) was significantly better than criteria I and II (*p* < 0.001, Table 3). According to the results of the diagnostic tests by criteria III, the negative predictive value was 90.91 % but the positive predictive value was 60.71 %. This finding of good negative predictive value explained that the low incidence of HGD/Tis/CA when the twisted elongated pattern of IPCL was not shown by NBI. Early detection of HGD/Tis/CA is directly related to less aggressive treatment and better prognosis. For those who are not suitable to receive a biopsy or that are not willing to undergo a surgical biopsy, NBI can be a promising, fast, and safe tool to provide addition important information regarding oral erythroplakia before surgical intervention.

To the best of our knowledge, the current study is the first to illustrate the correlation between the pathology and morphological pictures of NBI images in a large series of patients. The images of NBI are apparently subjective. Training to learn to observe the morphology from all angles and avoid the inadvertent light reflex from the mucus or debris of oral cavity is mandatory. In a study done by Puxeddu et al*.*, coupling of the NBI and Storz Professional Image Enhancement System (SPIES) with contact endoscopy for laryngeal and hypopharyngeal pathology a to magnify the vascular pattern of the lesions examined and reduce inter and intraobserver variations is a promising tool. The accuracy in the differential diagnosis between normal tissue and hyperplasia versus mild dysplasia and carcinoma is 97.6 % [34]. Further study of application of this system to the oral cavity mucosal lesions is warranted to improve the detection of pathological dysplasia and carcinoma. Lack of universal diagnostic standards for NBI microvascular morphology remains a challenge and large-scale prospective study is warranted for further validation of this tool. In addition, we are aware of some limitations of our study. First, the sample size is small due to the low incidence of oral erythroplakia and short time period for case collection. Second, the histopathological epithelial dysplasia is a spectrum, and currently no definite criteria are established to clearly cut this spectrum into low, intermediate, and high-grade. There may be a substantial interobserver and intraobserver variation in the assessment of the grade of epithelial dysplasia [14]. We attempted to reduce the above-mentioned variation by using immunohistochemical staining with Ki-67 mouse monoclonal antibody, in some of the cases, and by asking two pathologists to reach agreement on every case. The third limitation is the retrospective nature of the study. Large-scale, multi-center, or international cross-country research is required to achieve a more definite conclusion.

## Conclusion

Twisted elongated and destructive patterns of intraepithelial microvasculature of NBI images are crucial indicators for detecting high-grade dysplasia, carcinoma in situ, and invasive carcinoma in oral erythroplakia in the current study. The findings suggest that endoscopy with NBI may serve as a non-invasive procedure to provide adjunctive information in identifying HGD/Tis/CA in oral erythroplakia.

## Abbreviations

OSCC: Oral squamous cell carcinoma
NBI: Narrow-band imaging
HGD/Tis/CA: High-grade dysplasia carcinoma in situ, and invasive carcinoma
BWL: Broad-band white light
IPCL: Intraepithelial papillary capillary loop
OR: Odds ratio
CI: Confidence interval
Pearson’s *r*: the Pearson’s linear correlation coefficient
